# Relationship between Crystalline Lens Thickness and Shape and the Identification of Anterior Ocular Segment Parameters for Predicting the Intraocular Lens Position after Cataract Surgery

**DOI:** 10.1155/2019/3458548

**Published:** 2019-07-08

**Authors:** Tsukasa Satou, Kimiya Shimizu, Shuntaro Tsunehiro, Akihito Igarashi, Sayaka Kato, Manabu Koshimizu, Takahiro Niida

**Affiliations:** ^1^Department of Orthoptics and Visual Sciences, School of Health Sciences, International University of Health and Welfare, Tochigi, Japan; ^2^Sanno Hospital, Tokyo, Japan; ^3^International University of Health and Welfare, Tochigi, Japan

## Abstract

**Purpose:**

This study was performed to investigate the relationships among crystalline lens shape, actual intraocular lens (IOL) position, and crystalline lens thickness (LT), as measured by anterior segment optical coherence tomography (AS-OCT), and to determine anterior ocular segment parameters that predict postoperative IOL position.

**Methods:**

Seventy-nine eyes of 79 patients who underwent uneventful cataract surgery were enrolled. For crystalline lens preoperative anterior segment data, the LT, and anterior, equatorial, and posterior surface depths (ASD, ESD, and PSD, respectively) of crystalline lenses were quantitatively determined. For postoperative anterior segment data, the actual IOL position was quantified. Moreover, the following correlations were analyzed: LT with the ASD, ESD, PSD, and IOL position; IOL position with the ASD, ESD, and PSD; and refractive prediction error with the difference between the predicted postoperative anterior chamber depth (ACD) of the SRK/T formula and the IOL position, ASD, ESD, and PSD (each depth minus the predicted postoperative ACD of the SRK/T formula).

**Results:**

The LT was significantly correlated with the ASD (r = -0.65) and PSD (r = 0.41), whereas it was not correlated with the ESD or IOL position. The IOL position was significantly correlated with the ASD (r = 0.67), ESD (r = 0.72), and PSD (r = 0.74). The refractive prediction error was significantly correlated with the difference between the predicted postoperative ACD of the SRK/T formula and the IOL position (r = 0.65), ASD (r = 0.46), ESD (r = 0.54), and PSD (r = 0.58).

**Conclusions:**

The ESD and PSD obtained using AS-OCT were highly correlated with the IOL position and significantly correlated with the refractive prediction error. These findings suggest that the ESD and PSD may enhance the accuracy of actual IOL position prediction.

## 1. Introduction

Today, it is common knowledge that cataract surgery includes elements of refractive and presbyopic surgery, in addition to removal of the diseased tissue [1–5]. Implanting an intraocular lens (IOL) with the appropriate power calculation during cataract surgery affects patient satisfaction and leads to a successful surgery. Norrby et al. [6] demonstrated that preoperative prediction of the postoperative IOL position (i.e., postoperative anterior chamber depth [ACD]) contributed to the greatest proportion of IOL power prediction errors. The authors also reported that an estimated error of 1 mm in the postoperative ACD represents a refractive error of 1.44 D for an eye of average dimensions, a finding that has been validated based on ray tracing techniques [6]. Thus, improving the predictive accuracy of the IOL position is essential for reducing postoperative refractive error.

The postoperative ACD that is included in well-known, third-generation IOL power calculation formulas (SRK/T [7], Holladay 1 [8], and Hoffer Q [9]) does not reflect the true postoperative ACD in the anatomical sense, because it is calculated using thin lens formulas. The postoperative ACD is estimated via equations on the basis of axial length (AL) and mean keratometry (K) data; as such, it differs from the actual lens position. For the SRK/T and Holladay 1 formulas, the postoperative ACD is calculated as being deep in cases with a steep K and as being shallow in cases with a flat K, based on estimates made by utilizing the Pythagorean Theorem with K data [7, 8]. Newer formulas (Olsen [10, 11], Holladay 2 [12], and Barrett Universal II [13, 14]) require additional biometry parameters, especially those related to anterior segment anatomy, to better predict the postoperative ACD. Olsen T [10, 11] described the notion of using the C constant to predict the postoperative central IOL thickness position inside the preoperative lens bag, based on the preoperative lens thickness (LT) and ACD. However, the LT is known to increase with age; moreover, for a given increase in the overall LT, changes in the LT at the anterior and posterior segments of the lens may differ [15–18]. Importantly, the effects of LT on the difference between the postoperative actual IOL position and the predicted IOL position using the C constant have not been elucidated. Hence, investigations of the effects of LT on the crystalline lens shape, position, and postoperative actual IOL position are needed to verify the effect of LT on the predicted IOL position using the C constant and to improve prediction of the postoperative actual IOL position.

Notably, anterior segment optical coherence tomography (AS-OCT) can be employed to visualize the anterior segment, and thus shows promise as a helpful tool for clinically evaluating this region [19]. Recently, the CASIA2 device (TOMEY Corp., Nagoya, Japan)—an AS-OCT device with a swept-source laser wavelength of 1310 nm—was developed. Because the CASIA2 can perform deep measurements, including measurements of the anterior segment length, it may aid in the evaluation of crystalline lens shape and postoperative actual IOL position.

Here, our primary aim was to investigate the relationship between crystalline lens shape and LT, as well as the relationships between the predicted IOL position using the C constant and the postoperative actual IOL position and LT, by using the CASIA2 device. Our secondary aim was to determine anterior ocular segment parameters that may assist in the prediction of the actual IOL position.

## 2. Materials and Methods

### 2.1. Study Population

This was a prospective study conducted on patients who had undergone uneventful cataract surgery between April 2016 and March 2017 in Sanno Hospital, Tokyo, Japan. Participants were excluded from the study if they had a history of refractive surgery, intraocular surgery, or ocular pathology. The procedures used in this study were approved by the Institutional Review Board of Sanno Hospital (approval number 17-S-6) and conformed to the tenets of the Declaration of Helsinki. Written informed consent was obtained from each of the patients after he/she had been given an explanation of the study's purpose, risks, potential discomfort, and procedures.

All three surgeons involved in the study used a 2.8-mm postlimbal incision, 5.0-mm centered curvilinear capsulorhexis, and standard phacoemulsification with an in-the-bag IOL. The implanted IOL was a monofocal AQ110NV lens (STAAR Surgical, Monrovia, CA, USA) that consisted of three pieces. The IOL power was calculated using the SRK/T formula with data obtained through the IOLMaster 700 (Carl Zeiss Meditec AG, Jena, Germany).

### 2.2. Study Procedures and Calculations

**Figure 1:**
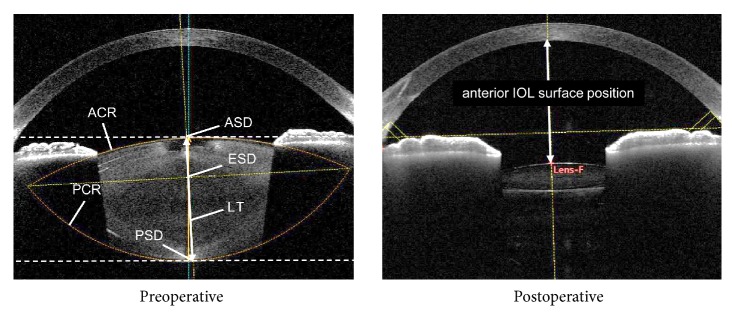
*Parameters measured with the CASIA2 device.* LT: crystalline lens thickness; ACR: anterior curvature radius; PCR: posterior curvature radius; ASD: anterior surface depth; ESD: equatorial surface depth; PSD: posterior surface depth.

We imaged patients' anterior segments using the CASIA2 device (TOMEY Corp.) both preoperatively and at 1 month postoperatively (Figure 1). The CASIA2 software presents the image (shape) corrected by a fixed refractive index at each segment. For preoperative anterior segment data, the anterior and posterior curvature radii (ACR and PCR, respectively) for the crystalline lens were automatically traced to fit a circle using the CASIA2 software and were quantified. The anterior surface depth for the crystalline lens (ASD) was automatically calculated as the distance between the anterior surface of the crystalline lens and the posterior surface of the cornea. In addition, the posterior surface depth for the crystalline lens (PSD) was calculated by adding the automatically calculated LT to the ASD. The equatorial surface for the crystalline lens was determined by drawing a line on the intersection point of the ACR and PCR (i.e., predictive equatorial surface), and the distance between that line and the posterior surface of the cornea was quantitatively determined as the equatorial surface depth for the crystalline lens (ESD). The pupil size was more than 4 mm with or without dilation.

For postoperative anterior segment data, the anterior IOL surface position of the actual implanted IOL was calculated as the distance between the anterior IOL surface position and the posterior surface of the cornea. Furthermore, the central IOL thickness position (anterior IOL surface position + IOL thickness/2) and posterior IOL surface position (anterior IOL surface position + IOL thickness) surface depth were calculated. If the crystalline lens axis was not identical to the optical axis, the straight-line distance was determined by using the crystalline lens axis. Postoperative refraction was measured with an autorefractor (TONOREF, Nidek Co., Ltd., Aichi, Japan).

### 2.3. C Constant

The C constant was calculated postoperatively, as recommended in the literature [11]. Olsen and Hoffman [11] described the notion of the C constant as a method to predict the postoperative position of the IOL inside the preoperative lens bag from the preoperative dimension and position of the lens as follows: IOL_C_ = ACD_pre_ + C ×  LT_pre_. The effective C constant was calculated after surgery as follows: C constant = (ACD_post_ + IOL thickness/2 - ACD_pre_)/LT_pre_. In these equations, the IOL_C_ is the central IOL thickness position, ACD_pre_ is the preoperative ACD, LT_pre_ is the preoperative LT, and ACD_post_ is the postoperative ACD.

In the current study, the mean value of the C constant of the AQ110NV implanted IOL was determined, and the predictive central IOL thickness position was calculated using the C constant (predicted IOL position using the C constant). The corneal thickness was excluded in the calculation of IOL_C_ and ACD.

### 2.4. Statistical Analysis

The correlations of the following parameters with LT were determined: age, ACR, PCR, ASD, ESD, PSD, central IOL thickness position, and predicted IOL position using the C constant. The correlations of the predicted IOL position using the C constant with ASD, ESD, and PSD were also determined, along with the correlations of the anterior IOL surface position with ASD, ESD, and PSD.

The correlations between the refractive prediction error and the difference between the predicted postoperative ACD of the SRK/T formula and the anterior IOL surface position, ASD, ESD, and PSD (each depth minus the predicted postoperative ACD of the SRK/T formula) were determined. The postoperative ACD of the SRK/T formula was calculated using the K value and AL, as measured by the IOLMaster 700, in accordance with a previous report [7]. The refractive prediction error was calculated as the eye refraction at 1 month postoperatively minus the predictive refraction, as determined by the SRK/T formula. In this formula, predictive refractions were calculated by optimizing the A constant.

The correlation coefficients among the ASD, ESD, and PSD were determined. Multiple linear regression analysis for predicting the postoperative anterior IOL depth based on the crystalline lens position variables was used to determine the standard partial regression coefficient and multiple regression coefficient of determination R^2^ values.

The correlation analyses were performed using Pearson's product moment correlation coefficient. The p-values were corrected by Bonferroni correction; the level of statistical significance was set at p < 0.05. Statistical analyses were performed with the commercially available statistical software SPSS (version 24.0; IBM Corp., Armonk, NY, USA).

## 3. Results

The demographic data and ocular biometric parameters of the patients are presented in Table 1; 79 eyes of 79 patients (27 men and 52 women) were enrolled; the patients ranged in age from 26 to 86 years, with a mean (± standard deviation) age of 69 ± 11 years. The mean central IOL thickness position was 4.55 mm, whereas the mean predicted IOL position using the C constant was 4.54 mm, and the ESD was 4.24 mm. The mean predicted postoperative ACD based on the SRK/T formula was 5.68 mm.

**Figure 2:**
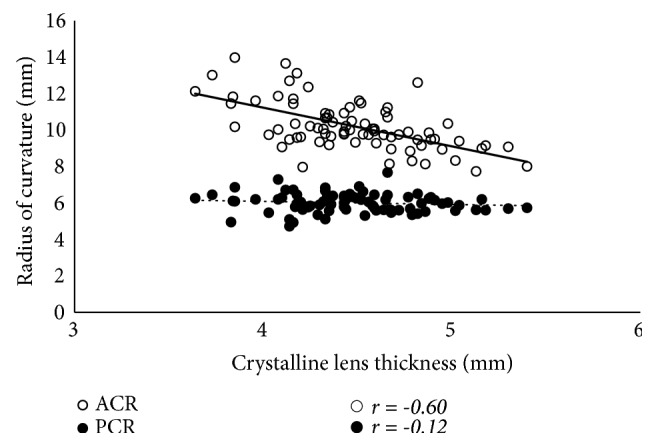
*Correlations between the lens thickness and the anterior and posterior curvature radii for the crystalline lens*. ACR: anterior curvature radius for the crystalline lens; PCR: posterior curvature radius for the crystalline lens.

**Figure 3:**
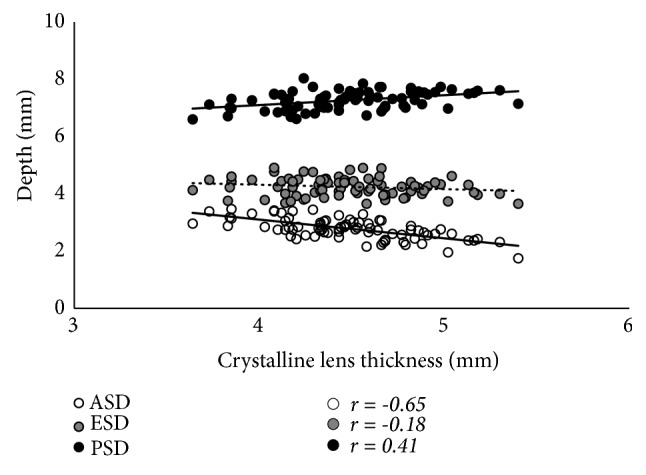
*Correlations between the lens thickness and the anterior, equatorial, and posterior surface depths for the crystalline lens*. ASD: anterior surface depth for the crystalline lens; ESD: equatorial surface depth for the crystalline lens; PSD: posterior surface depth for the crystalline lens.

**Figure 4:**
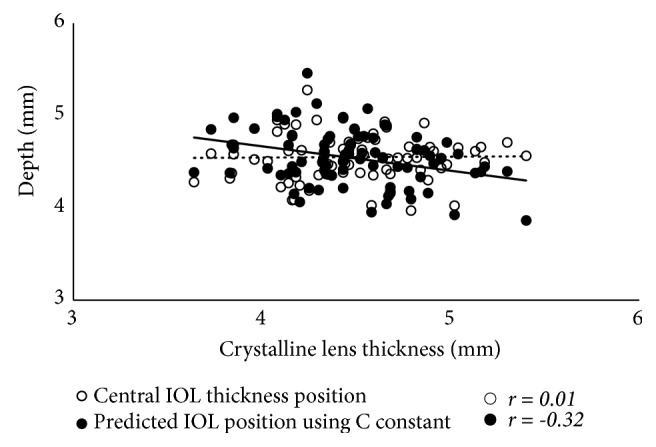
*Correlations between the lens thickness and the central IOL thickness position and predicted IOL position using the C constant*. IOL: intraocular lens.

**Figure 5:**
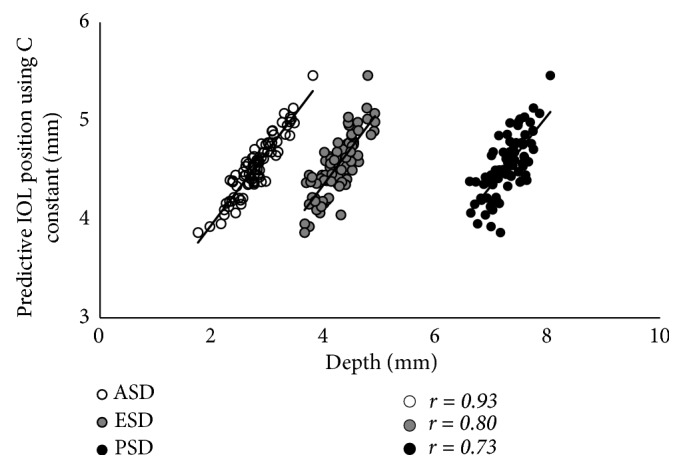
*Correlations between the anterior, equatorial, and posterior surface depths for the crystalline lens and the predicted IOL position using the C constant*. ASD: anterior surface depth for the crystalline lens; ESD: equatorial surface depth for the crystalline lens; PSD: posterior surface depth for the crystalline lens; IOL: intraocular lens.

**Figure 6:**
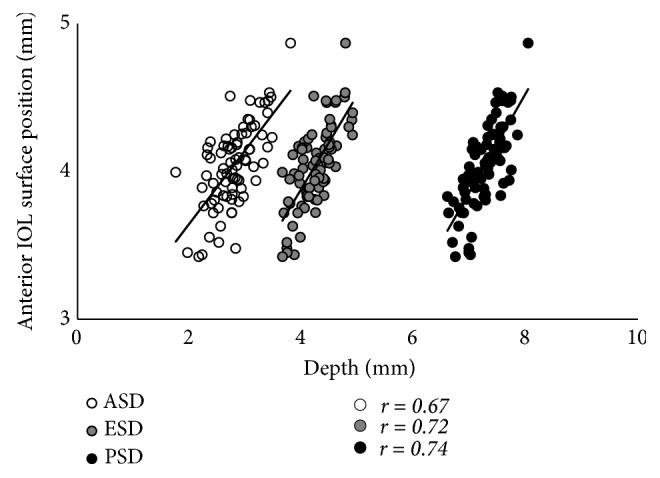
*Correlations between the anterior, equatorial, and posterior surface depths for the crystalline lens and the anterior IOL surface position*. ASD: anterior surface depth for the crystalline lens; ESD: equatorial surface depth for the crystalline lens; PSD: posterior surface depth for the crystalline lens; IOL, intraocular lens.

**Figure 7:**
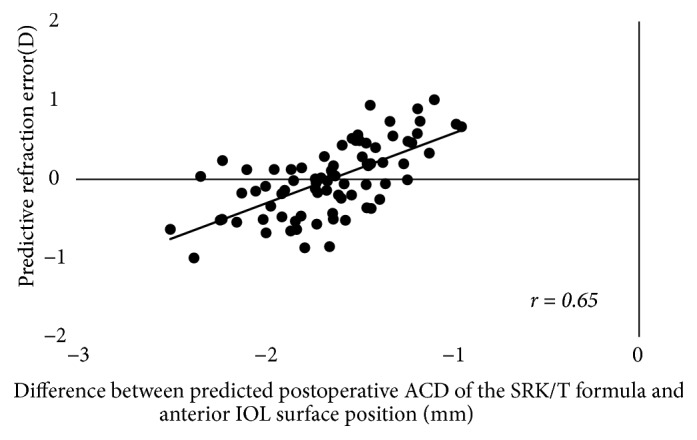
*Correlations of the refractive prediction error with the difference between the predicted postoperative ACD of the SRK/T formula and the anterior IOL surface position*. ACD: anterior chamber depth; IOL: intraocular lens.

**Figure 8:**
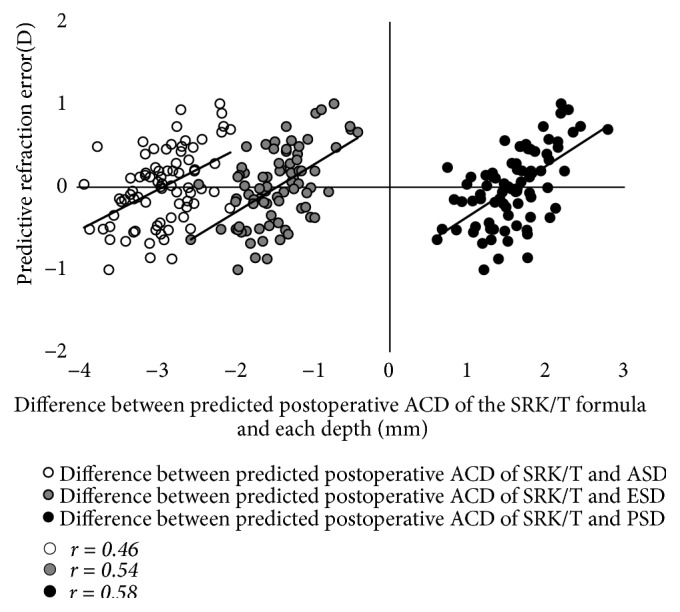
*Correlations of the refractive prediction error with the difference between the predicted postoperative ACD of the SRK/T formula and the anterior, equatorial, and posterior surface depths for the crystalline lens*. ACD: anterior chamber depth; ASD: anterior surface depth; ESD: equatorial surface depth; PSD: posterior surface depth.

The results of the simple correlation analyses are shown in Table 2. The LT was significantly correlated with age, as well as with the ACR, ASD, and PSD (p < 0.001 for all), but was not correlated with the PCR or ESD (Figures 2 and 3). The LT was significantly correlated with the C constant (p < 0.001) and predicted IOL position using the C constant (p = 0.033), but was not correlated with the central IOL thickness position (Figure 4). In addition, these correlative relationships did not substantially differ in terms of partial correlation coefficients, when age was set as the control variable. The predicted IOL position using the C constant was significantly correlated with the ASD, ESD, and PSD (p < 0.001 for all) (Figure 5). Furthermore, the anterior IOL surface position was significantly correlated with the ASD, ESD, and PSD (p < 0.001 for all) (Figure 6). The refractive prediction error was significantly correlated with the difference between the anterior IOL surface position and the predicted postoperative ACD of the SRK/T formula (p < 0.001) (Figure 7). The refractive prediction error was also significantly correlated with the difference between the predicted postoperative ACD of the SRK/T formula and the ASD, ESD, and PSD (p < 0.001 for all) (Figure 8).

The simple correlation coefficients for the ASD, ESD, and PSD are presented in Table 3. The ASD was poorly correlated with the PSD.  Table 4 presents the results of the multiple regression analysis for predicting the anterior IOL surface position based on the ASD, ESD, and PSD. The ESD was strongly correlated with the anterior IOL surface position according to the simple correlation coefficient analysis; however, the standard partial regression coefficient of the ESD in the multiple regression analysis for predicting the anterior IOL surface position was 0.17, and thus the contribution of the ESD was less than the contributions of the ASD and PSD for predicting the anterior IOL surface position.

## 4. Discussion

In the present study, we determined the relationships among the crystalline lens shape, IOL position, and LT using the CASIA2 device. Although our findings do not show temporal changes, they suggest that as the LT increases, the anterior surface of the crystalline lens steepens and moves anteriorly (ASD = -0.66 × LT + 5.72), whereas the posterior surface moves slightly posteriorly (PSD = 0.34 × LT + 5.72). These results are concordant with those in previous reports [15–18] showing that the crystalline lens shape changes with age. In addition, the correlative relationships did not differ greatly in terms of the partial correlation coefficient when age was set as the control variable; this suggests that the age-related increase in LT may present the changes in crystalline lens shape that we observed in this study.

The effects that increases in LT have on the ESD and IOL position have not been previously clarified; in the present study, the ESD and IOL position were not correlated with the LT. Kasthurirangan et al. [20] reported that the ciliary body depth (axial distance between the anterior cornea and a line joining the innermost ciliary body tips) did not change with age. The zonule of Zinn has its origin at the ciliary body tips and extends to the equatorial segment of the crystalline lens. Based on these observations, we suspected that, like the ciliary body depth, the ESD might remain nearly unaffected by aging. Indeed, we found that the ESD was not correlated with the LT. Furthermore, neither the IOL position nor the ESD were correlated with the LT, and the central IOL thickness position was slightly deeper than the ESD. The haptic angle in a three-piece AQ110NV IOL implant is designed to lean 10° toward the anterior. Therefore, one explanation for our results might be that the IOL haptics were set in the supporting section of the crystalline lens capsule (i.e., equatorial section) and the IOL was located slightly posteriorly.

Previously, Olsen et al. [11] developed the C constant to predict the postoperative position of the IOL from the preoperative thickness and position of the crystalline lens. The C constant varies depending on the IOL design, but typically has a value of approximately 0.40. In this study, the mean value of the C constant was 0.39, and it was used to predict the IOL position. The predicted IOL position using the C constant was shallow when there was an increase in the LT, while the central IOL thickness position was not correlated with the LT. In a study by Plat J et al. [21] in which ocular optical biometry measurements were obtained using a Lenstar LS900 (Haag-Streit, Bern, Switzerland) before and after cataract surgery, the actual IOL position was not correlated with the LT, and our results support this finding. As described above, the anterior surface of the crystalline lens moved anteriorly, whereas the posterior surface moved slightly posteriorly, with an increase in the LT. As a result, the center position of the crystalline lens (i.e., 0.50 in the C constant) shifts anteriorly; thus, the predicted IOL position using the C constant is calculated as being more anterior when there is an increase in the LT. This suggests that the predicted IOL position using the C constant is affected by the LT, which may increase the difference between the predicted and actual lens positions. However, the calculated C constant based on the actual IOL position was positively correlated with the LT, and as such, it might be improved by adjusting the LT.

Based on the abovementioned findings, we further examined the relationships between the anterior IOL surface position and the ASD, ESD, and PSD. We found that the anterior IOL surface position was correlated closely with the PSD and ESD. Our results were equivalent to the correlation identified previously by Goto et al. [19] between the postoperative ACD and the angle-to-angle depth. The correlation between the anterior IOL surface position and the ASD was not as strong as that between the anterior IOL surface position and the PSD or ESD. This may be because the ASD is more strongly affected by an increase in the LT than by an increase in the PSD; hence, the ASD was poorly correlated with the PSD. In contrast, the predicted IOL position using the C constant (geometrically calculated from the ACD and LT) was significantly correlated with the ASD (R^2^ = 0.86) and showed a lack of agreement with the actual IOL position. Plat J et al. [21] reported that the anterior segment depth (PSD in this study) was correlated with the actual IOL position after cataract surgery. In contrast, the correlation between the ESD and IOL position has not been clarified; the results of our study suggest that the ESD may be a useful parameter for predicting the postoperative IOL position.

The present study also yielded evidence supporting that the PSD and ESD may be able to reduce the refractive error after cataract surgery. As previously mentioned, using the SRK/T formula, the predicted postoperative ACD can be estimated with the Pythagorean Theorem using K data [7] and is calculated as being deeper when there is a steep K and shallower when there is a flat K. Therefore, a shallower IOL position than the predicted postoperative ACD according to the SRK/T formula results in a greater shift to myopia relative to the predicted refraction. Herein, we determined the correlations between the refractive prediction error and the difference between the predicted postoperative ACD of the SRK/T formula and the ASD, ESD, PSD, and anterior IOL surface position. We found that the correlation coefficient for the relationship between the refractive prediction error and the difference between the predicted postoperative ACD of the SRK/T formula and the anterior IOL surface position was the highest, at 0.65. The PSD had the second highest correlation coefficient (r = 0.58), followed by the ESD (r = 0.54). Thus, the PSD and ESD are associated with the predictive refraction error after cataract surgery when using the SRK/T formula; we speculate that by employing the SRK/T formula, we might be able to select the IOL power in anticipation of the predictive refraction error after cataract surgery. Moreover, if we can accurately predict the postoperative IOL position from the PSD or ESD and accurately measure the true AL and true K, we may be able to reduce the postoperative predictive refraction error by utilizing optical simulation. Martinez-Enriquez et al. [22] reported that the lens equatorial plane position (distance between the anterior lens apex and the position of the equatorial plane) was exceptionally valuable for estimating the IOL position when combined with the LT, and our results support this finding.

In the multiple regression analysis for predicting the anterior IOL surface position, the standard partial regression coefficient of ESD was lower than those of ASD and PSD, which showed different results from the simple correlation coefficient. A correlation between ASD, ESD, and PSD may be responsible for these results. Since ESD has a strong correlation with the anterior IOL surface position in the simple correlation coefficient, it cannot be concluded that ESD has a lower contribution to the anterior IOL surface position. In addition, a note should be made of the measurement method of ESD. The ESD is directly affected by the measurement error of the partially measured ACR and PCR of the crystalline lens, because the equatorial surface of the crystalline lens is determined by drawing a line along the intersection point of the ACR and PCR, as previously described. Thus, the predictive equatorial surface is distinct from the actual equatorial surface of the crystalline lens in its anatomical location. In an individual with a small pupil size, the extrapolated shape may be obviously different from the actual shape of the lens. In contrast, it is unlikely that measurement error affected the PSD, because the PSD can be measured directly from the obtained image. Nevertheless, as the ESD lies between the ASD and PSD, a moderate correlation is ensured, even in cases of inaccurate ESD measurements. If a new index ESD can be measured accurately under fixed conditions, it might most contribute the postoperative IOL position in multiple regression analysis. Future studies are warranted to clarify the relationship between the ESD created by the CASIA2 device and the actual equatorial surface of the crystalline lens in its anatomical location and to determine the effects of pupil size on the ESD.

Limitations of the present study include its small sample size, the inclusion of only one IOL implant type, and an AL that was biased toward long eyes. Thus, a larger study is needed to more clearly indicate the utility of the ESD and PSD in IOL power calculations.

## 5. Conclusion

This study found that the ESD and PSD obtained using the new CASIA2 device were highly correlated with the IOL position and were significantly correlated with the refractive prediction error. In addition, neither the ESD nor the IOL position was affected by the LT; a new index ESD might have the potential to predict the postoperative IOL position more accurately.

## Figures and Tables

**Table 1:** Demographic data and mean ocular biometric parameters.

Parameter	Mean ± SD (range)
Eyes (n)	79
Patients (n)	79
Age (years)	69 ± 11 (26–86)
Females, n (%)	50 (65.8%)
Implanted IOL	AQ110NV
IOL power (D)	19.4 ± 3.53 (10.0–25.5)
Axial length (mm)	24.60 ± 1.51 (21.74–28.47)
Predictive refraction by SRK/T for after surgery (D)	-1.51 ± 0.84 (-3.10–-0.30)
Spherical equivalent value of postoperative refraction (D)	-1.51 ± 1.03 (-3.38–0.38)
Prediction error (D)	0.00 ± 0.45 (-1.00–1.01)
Predicted postoperative ACD of SRK/T formula (mm)	5.68 ± 0.41 (4.86–6.98)
C constant	0.39 ± 0.04 (0.30–0.52)
Predicted IOL position using C constant (mm)	4.54 ± 0.30 (3.87–5.46)
Preoperative crystalline lens parameters	
Anterior curvature radius (mm)	10.21 ± 1.32 (7.73–13.97)
Posterior curvature radius (mm)	6.01 ± 0.53 (4.72–7.67)
Lens thickness (mm)	4.48 ± 0.37 (3.64–5.40)
Anterior surface depth (mm)	2.79 ± 0.38 (1.75–3.80)
Equatorial surface depth (mm)	4.24 ± 0.32 (3.65–4.91)
Posterior surface depth (mm)	7.27 ± 0.32 (6.60–8.04)
Postoperative IOL depth parameters	
Anterior IOL surface position (mm)	4.04 ± 0.28 (3.42–4.87)
Central IOL thickness position (mm)	4.55 ± 0.25 (3.97–5.28)
Posterior IOL surface position (mm)	5.06 ± 0.22 (4.51–5.69)

SD: standard deviation; IOL: intraocular lens; ACD: anterior chamber depth.

**Table 2:** Correlations and determination coefficients of the ocular biometric parameters.

Variable	Correlation and determination coefficients (partial correlation coefficients) with crystalline lens thickness	p value
Age	r = 0.53, R^2^ = 0.28	<0.001
Anterior curvature radius for crystalline lens	r = -0.60, R^2^ = 0.36 (r = -0.57)	<0.001
Posterior curvature radius for crystalline lens	r = -0.12, R^2^ = 0.01 (r = -0.14)	1.000
Anterior surface depth for crystalline lens	r = -0.65, R^2^ = 0.42 (r = -0.61)	<0.001
Equatorial surface depth for crystalline lens	r = -0.18, R^2^ = 0.03 (r = -0.17)	1.000
Posterior surface depth for crystalline lens	r = 0.41, R^2^ = 0.17 (r = 0.32)	0.002
Central IOL thickness position	r = 0.01, R^2^ = 0.00 (r = 0.02)	1.000
C constant	r = 0.55, R^2^ = 0.30 (r = 0.54)	<0.001
Predicted IOL position using C constant	r = -0.32, R^2^ = 0.10 (r = -0.32)	0.033
	Correlation and determination coefficients with predicted IOL position using C constant	
Anterior surface depth for crystalline lens	r = 0.93, R^2^ = 0.86	<0.001
Equatorial surface depth for crystalline lens	r = 0.80, R^2^ = 0.64	<0.001
Posterior surface depth for crystalline lens	r = 0.73, R^2^ = 0.53	<0.001
	Correlation and determination coefficients with anterior IOL surface position	
Anterior surface depth for crystalline lens	r = 0.67, R^2^ = 0.45	<0.001
Equatorial surface depth for crystalline lens	r = 0.72, R^2^ = 0.52	<0.001
Posterior surface depth for crystalline lens	r = 0.74, R^2^ = 0.55	<0.001
	Correlation and determination coefficients with predictive refraction error	
Difference between predicted postoperative ACD of SRK/T formula and anterior IOL surface position	r = 0.65, R^2^ = 0.42	<0.001
Difference between predicted postoperative ACD of SRK/T formula and anterior surface depth for crystalline lens	r = 0.46, R^2^ = 0.21	<0.001
Difference between predicted postoperative ACD of SRK/T formula and equatorial surface depth for crystalline lens	r = 0.54, R^2^ = 0.29	<0.001
Difference between predicted postoperative ACD of SRK/T formula and posterior surface depth for crystalline lens	r = 0.58, R^2^ = 0.34	<0.001

IOL: intraocular lens; ACD: anterior chamber depth.

**Table 3:** The simple correlation coefficients among the anterior, equatorial, and posterior surface depths for the crystalline lens.

	Anterior surface depth	Equatorial surface depth	Posterior surface depth
Anterior surface depth	-	r = 0.71, p < 0.001	r = 0.43, p < 0.001
Equatorial surface depth	r = 0.71, p < 0.001	-	r = 0.64, p < 0.001
Posterior surface depth	r = 0.43, p < 0.001	r = 0.64, p < 0.001	-

**Table 4:** Multiple regression analysis for predicting the anterior intraocular lens surface position from the crystalline lens position variables.

Variable	Partial regression coefficient	Standard partial regression coefficient	Simple correlation coefficient
Anterior surface depth	0.25	0.33	0.67
Equatorial surface depth	0.15	0.17	0.72
Posterior surface depth	0.44	0.49	0.74
Constant	-0.47		
	Multiple coefficient of determination R^2^ = 0.71		

## Data Availability

The minimal data set used to support the findings of this study is included within the supplementary information file (available here).

## Supplementary Materials

Supplementary Materials See S1 in the Supplementary Materials for minimum data set.
